# Role of HCN channels in the functions of basal ganglia and Parkinson’s disease

**DOI:** 10.1007/s00018-024-05163-w

**Published:** 2024-03-13

**Authors:** Zeng-Xin Qi, Qi Yan, Xiu-Juan Fan, Jian-Ya Peng, Hui-Xian Zhu, Yi-Miao Jiang, Liang Chen, Qian-Xing Zhuang

**Affiliations:** 1 Department of Physiology, School of Medicine, Nantong University, 19 Qixiu Road, Nantong, 226001 Jiangsu China; 2 Department of Neurosurgery, Huashan Hospital, Shanghai Medical College, Fudan University, Shanghai, 200030 China; 3 National Center for Neurological Disorders, Shanghai, 200030 China; 4 Shanghai Key Laboratory of Brain Function Restoration and Neural Regeneration, Shanghai, 200030 China; 5 State Key Laboratory of Medical Neurobiology and MOE Frontiers Center for Brain Science, Institutes of Brain Science, Fudan University, Shanghai, 200030 China

**Keywords:** Parkinson’s disease, HCN channel, Hyperpolarization-activated current, Firing rate, Firing pattern, Basal ganglia

## Abstract

Parkinson's disease (PD) is a motor disorder resulting from dopaminergic neuron degeneration in the substantia nigra caused by age, genetics, and environment. The disease severely impacts a patient’s quality of life and can even be life-threatening. The hyperpolarization-activated cyclic nucleotide-gated (HCN) channel is a member of the HCN1-4 gene family and is widely expressed in basal ganglia nuclei. The hyperpolarization-activated current mediated by the HCN channel has a distinct impact on neuronal excitability and rhythmic activity associated with PD pathogenesis, as it affects the firing activity, including both firing rate and firing pattern, of neurons in the basal ganglia nuclei. This review aims to comprehensively understand the characteristics of HCN channels by summarizing their regulatory role in neuronal firing activity of the basal ganglia nuclei. Furthermore, the distribution and characteristics of HCN channels in each nucleus of the basal ganglia group and their effect on PD symptoms through modulating neuronal electrical activity are discussed. Since the roles of the substantia nigra pars compacta and reticulata, as well as globus pallidus externus and internus, are distinct in the basal ganglia circuit, they are individually described. Lastly, this investigation briefly highlights that the HCN channel expressed on microglia plays a role in the pathological process of PD by affecting the neuroinflammatory response.

## Introduction

Parkinson's disease (PD) is among the most frequently occurring central nervous system disorders, with an increasing incidence rate globally. It is characterized by changes in the function of the entire basal ganglia network due to the slow and progressive degeneration of the dopaminergic neurons in the brain’s substantia nigra pars compacta (SNpc) [1–4]. The main clinical manifestations of PD patients are persistent tremors, bradykinesia, muscle rigidity, and postural instability [5, 6]. PD is associated with various risk factors, including aging, genetics, and environmental exposure, with aging being the most significant [7, 8]. Aging impacts the immune system, with immune stress being an important consequence. Postmortem studies of the SNpc in individuals without PD reveal moderate pathological changes, including mild mitochondrial dysfunction, dysregulated calcium and iron levels, and antioxidant deficiencies. These changes are more prevalent in this brain region than in other areas of similar age [9, 10]. This correlation suggests that age-related biomolecular changes in PD-prone brain regions, specifically the SNpc, increase the risk of PD onset. In addition to aging, the risk of developing PD is elevated by both environmental exposure and genetic factors. Studies have shown that pesticide exposure and traumatic brain injury are among the environmental factors that can influence the incidence of PD [8, 11, 12]. Moreover, approximately 5–10% of PD cases are familial, resulting from genetic mutations, and genome-wide association studies (GWAS) have identified genes that contain common genetic variants that increase susceptibility to PD [13, 14].

The HCN1-4 gene family encodes the hyperpolarization-activated cyclic nucleotide-gated channel (HCN) which transmits a hyperpolarization-activated current (Ih) that has unique effects on neuronal excitability and rhythmic activity [15–17]. PD murine models have revealed that the function of HCN channels, widely distributed in the SN, striatum, subthalamic nucleus (STN), and pallidum of the basal ganglia, is abnormal, indicating their contribution to underlying causes of PD symptoms [18–22]. The suppression of the Ih current in SN dopaminergic neurons is closely associated with the degeneration of these neurons in drug-induced and gene-modified rat PD model [23]. In addition, MitoPark mice are a PD model characterized by mutations in genes affecting dopaminergic neuronal function. These mutations cause mitochondrial dysfunction and the formation of intracellular inclusion bodies similar to Lewy’s bodies and have reduced Ih current density before the onset of PD symptoms [24]. Moreover, in the 1-methyl-4-phenyl-1,2,3,6-tetrahydropyridine (MPTP)-induced mouse model of PD, the active metabolite, MPP^+^, penetrates dopaminergic neuron mitochondria via dopamine transporters, inhibiting mitochondrial complex 1 activity, leading to oxidative damage and ATP synthesis blockade. ATP consumption opens ATP-sensitive potassium channels and ultimately inhibits the Ih current [25].

HCN channels are a promising candidate for drug development, although their pharmacological efficacy is relatively modest compared to other voltage-gated and ligand-gated ion channels. Research have demonstrated the efficacy of ivabradine, ZD7288, and cesium in inhibiting HCN channels in vivo in animal models [18, 21, 26, 27]. However, only ivabradine is currently approved for human use as the sole HCN channel inhibitor, which blocks all four HCN isoforms equally. Ivabradine, approved for treating chronic coronary artery disease (CAD) and chronic heart failure (CHF), act as a heart rate-lowering agent. Furthermore, preclinical research indicates that ivabradine may possess antiarrhythmic properties [28, 29]. The involvement of HCN subtypes in the central nervous system and PD pathology underscores the need for novel HCN-targeted drugs as a therapeutic approach for PD. HCN1-4 channels exhibit significant pharmacological differences, distinct functional roles, and varied tissue distributions [19–21]. As a result, the possibility of targeting specific tissues and subtypes in PD pathology presents an attractive avenue for developing PD therapy.

Recent studies have extensively explored the role of HCN channels in neurological disorders, such as epilepsy, neuropathic pain, affective disorders, PD, Alzheimer's disease (AD), amyotrophic lateral sclerosis (ALS), and spinal muscular atrophy (SMA). They also explore the potential of HCN channels as therapeutic targets and ways to design drugs to act on specific isoforms [15, 30]. Based on recent research conducted by our laboratory and others, this review aims to investigate the significance of HCN channels in neurons of the basal ganglia nuclei [17–21, 27, 31–34]. Specifically, this review aims to examine alterations in the expression of these channels within basal ganglia nuclei neurons and how they impact neuronal electrical activity in both wide-type and PD animal models, with the ultimate goal of investigating the involvement of HCN channels in PD pathogenesis. This review first addresses the composition, expression, distribution, and function of HCN channels. Then, it delves into the distribution and functions of HCN channel isoforms in various basal ganglia nuclei, focusing on their altered expression in PD and their effects on motor and non-motor symptoms. We emphasize the involvement of HCN channels in PD movement disorders due to their effects on neuron firing rate and pattern. Lastly, we examine the role of HCN channels in PD-associated neuroinflammation.

## Basic features of HCN channels

**Fig. 1:**
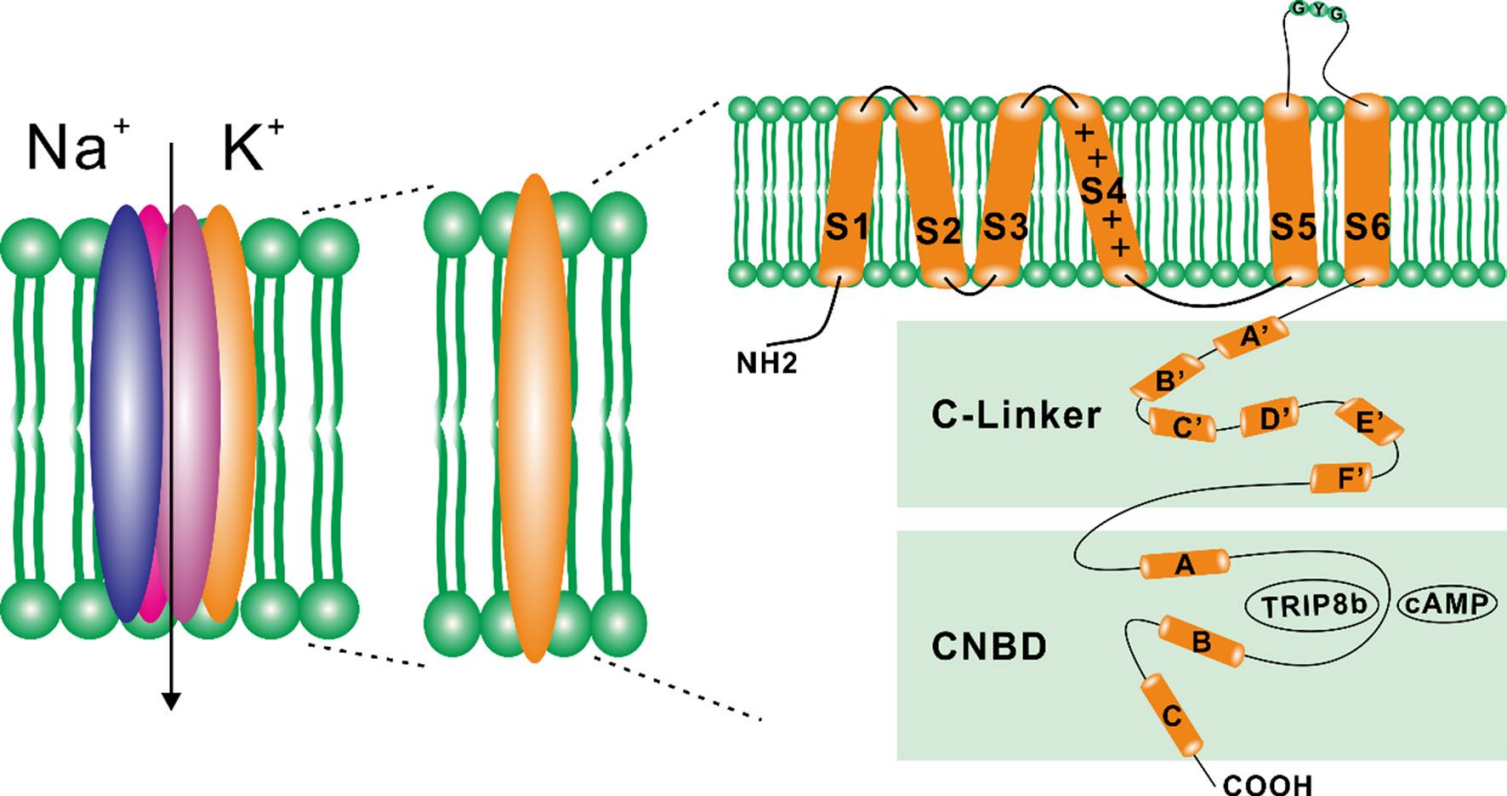
Probable structure of HCN channels. HCN channels function as tetramers, which can be homologous or heterologous (left). Each HCN subunit comprises six transmembrane segments: the NH2 terminal, the voltage sensor (S4), the pore region between S5 and S6, and the COOH terminal (right). The pore region contains the selectivity filter that carries the glycine-tyrosine-glycine amino-acid (GYG) sequence. The channel’s COOH terminal domain comprises the C-linker (comprises six α-helices designated A’ to F’) and the cyclic nucleotide-binding domain (CNBD) located after the C-linker domain. CNBD consists of alpha-helices **A**–**C** and a beta-roll between helices **A** and **B**. TRIP8b and cAMP regulate CNBD

In mammals, HCN channels are encoded by four genes (*Hcn1-4*), each producing a channel protein, namely HCN1, HCN2, HCN3, and HCN4 [35, 36]. HCN channel is a tetrameric structure composed of four subunits, and each subunit has four major structural modules, including a transmembrane voltage-sensing domain (S1-S4), a transmembrane pore-forming domain (S5-S6), a cytosolic C-link, and a cyclic nucleotide-binding domain (CNBD). In the membrane, the S4 helix carrying positive charges acts as the voltage sensor and shifts outward during membrane depolarization and inward during hyperpolarization. The voltage-sensing and pore-forming domains are covalently connected via the S4-S5 linker. The pore-forming domain has the glycine-tyrosine-glycine amino-acid (GYG) motif between S5 and S6, creating the ion selectivity filter. Following S6, there are 80 residue C-linkers composed of six α-helices (A'-F') and CNBD, which comprises three α-helices (A-C) and a β-roll between the A- and B-helix. The C-linker and CBND are collectively known as "cAMP sensing domains" or "tetratricopeptide repeat-containing Rab8b-interacting protein (TRIP8b) sensing domains". These molecules critically modulate the positive shift of voltage-dependent HCN channel activation by cAMP or TRIP8b (Fig. 1) [22, 37, 38].

Studies have indicated that there are three processes involved in the opening of the HCN channels: voltage-sensing domain activation, spreading of the activation energy from the voltage-sensing to the pore-forming domain, and a structural rearrangement of the pore-forming domain to open the pore gate, allowing the ions to pass through the membrane [39, 40]. Recently, it was revealed that the S4-S5 linker between the voltage-sensing and pore domains does not modulate the ligand-dependent gating or hyperpolarization-dependent activation. In contrast, the pore domain's voltage-sensing domain and the hyperpolarization voltage reduce this self-inhibition, opening the pore [41]. However, more studies are required to understand the opening mechanism of the HCN channel. HCN channels comprise tetrameric structures, forming four distinct tetramer subtypes with different activation kinetics *in-vivo*. HCN1 has the fastest activation speed, and HCN4 has the lowest activation speed, whereas HCN2 and HCN3 have a constant activation time between HCN1 and HCN4. These four subtypes exhibit different sensitivities to cAMP, where HCN2 and HCN4 are strongly regulated, while HCN1 and HCN3 indicate weak regulation (Table 1). In addition, in normal tissue, HCN channel subtypes aggregate into heterotetrameric structures, forming functional HCN channels. This combination enhances the range of HCN channels and their unique functions within the central nervous system [37, 42–44].

**Table 1:** Half-activation potentials (V_1/2_) and activation time constants (τ_on_) measured at − 120 mV, − 100 mV*, or − 110.5 mV^#^ for the four subtypes of the HCN channel

Subtypes	V_1/2_(mV)	τ_on_ (ms)	Cell type	References
HCN1 (Clone)	− 72.8 ± 0.2		Sinoatrial node cells	Saponaro et al. 2018 [17]
	− 80.5 ± 0.6		HEK293 cells	Chen et al. 2005 [49]
	− 79.3 ± 9.1	31.8 ± 4.8	*X. laevis* oocytes	Meng et al. 2011 [50]
	− 69.5 ± 3.3	67.0 ± 16.0*	HEK293 cells	Stieber et al. 2005 [51]
HCN2 (Clone)	− 93.7 ± 0.3		Sinoatrial node cells	Saponaro et al. 2018 [17]
	− 98.6 ± 0.6		HEK293 cells	Chen et al. 2005 [49]
	− 100.7 ± 1.3	217.8 ± 40.8	*X. laevis* oocytes	Meng et al. 2011 [50]
	− 95.6 ± 3.8	562.0 ± 198.0*	HEK293 cells	Stieber et al. 2005 [51]
HCN3 (Clone)	− 77.0 ± 5.3	1244.0 ± 198.0*	HEK293 cells	Stieber et al. 2005 [51]
HCN4 (Clone)	− 102.8 ± 0.3	2000 ± 200	Sinoatrial node cells	Saponaro et al. 2018 [17]
		1357 ± 227.9	*X. laevis* oocytes	Meng et al. 2011 [50]
	− 100.5 ± 3.3	5686.0 ± 2234.0*	HEK293 cells	Stieber et al. 2005 [51]
HCN1-4	− 91.7 ± 1.3	145.2 ± 8.9	EPN PV neurons	Peng et al. 2023 [18]
	− 95.8 ± 0.9		STN neurons	Zhuang et al. 2018 [19]
	− 95.5 ± 1.1		Vestibular ganglion neurons	Bronson and Kalluri 2023 [52]
	− 107.6 ± 2.7		LHb neurons	Good et al. 2013 [53]
	− 79.0 ± 1.0	95.6 ± 7.0^#^	MSO ventral neurons (P18)	Baumann et al. 2013 [54]
	− 87.0 ± 2.0	191.3 ± 28.1^#^	MSO dorsal neurons (P18)	Baumann et al. 2013 [54]
	− 76.0 ± 3.0	78.0 ± 3.6^#^	MSO ventral neurons (P22)	Baumann et al. 2013 [54]
	− 81.0 ± 2.0	109.6 ± 18.7^#^	MSO dorsal neurons (P22)	Baumann et al. 2013 [54]
	− 101.9 ± 1.6	815.0 ± 170.0	Small-sized DRG neurons	Tu et al. 2004 [55]
	− 99.5 ± 0.9	646.0 ± 64.0	Medium-sized DRG neurons	Tu et al. 2004 [55]
	− 97.3 ± 1.2	266.0 ± 17.0	Large-sized DRG neurons	Tu et al. 2004 [55]
	− 83.9	190.4 ± 23.1	BCs of the hippocampus	Aponte et al. 2006 [56]

*HEK293* human embryonic kidney 293, *EPN* entopeduncular nucleus, *STN* subthalamic nucleus, *PV* parvalbumin, *LHb* lateral habenular nucleus, *MSO* medial superior olive, *DRG* dorsal root ganglion, *BCs* basket cells.

The HCN channel mediates a membrane potential that generates an inward rectifying Ih current. This current exhibits rectification properties characterized by a higher conductance in the negative voltage range [17, 32]. The HCN channel’s current characteristic was initially discovered in neurons and cardiac sinoatrial node cells in the late 1970s. It was widely studied in the biomedical field during the early 1980s. Later, it was also found in rod and pyramidal cells in the CA1 region of the hippocampus [22, 45]. The HCN channel has unique ion selectivity and gating characteristics. For instance, it selectively and interdependently permeates Na^+^ and K^+^ simultaneously, and K^+^ permeability is 3 to 5 times larger than Na^+^ [22]. HCN channels are expressed in various brain regions, primarily activated neurons, when the resting membrane potential (RMP) falls below -50 mV. Inhibiting the Ih current results in RMP hyperpolarization and increased membrane impedance. As a result, the impact of modified HCN channel expression on neuronal excitability is determined by the interplay between RMP and resistance [22]. Rest potential hyperpolarization reduces neuronal excitability by moving them away from the firing threshold. In contrast, depolarization of the resting potential increases the input resistance of neurons and decreases the current required to depolarize cells, thereby increasing excitability [15, 27, 33]. However, research shows that HCN channel activity significantly affects the subliminal conductance of other co-expressed ion channels in specific neurons. Therefore, Ih’s overall effect depends on the unique combination and relative density of subthreshold conductance from all ion channel types within a neuron class, potentially modulating neuronal excitability in varied situations [15, 37, 46]. HCN channel-mediated Ih currents serve to maintain normal body functions, such as rhythmic and pacemaker activities, dendritic integration, synaptic transmission, spatial working memory, water and electrolyte homeostasis, perception, neuronal proliferation, and sensory signaling [16, 47]. However, abnormal HCN channel expression under pathological conditions can cause neurological dysfunction [22, 48].

## Basal ganglia HCN channel expression and PD symptoms

**Fig. 2:**
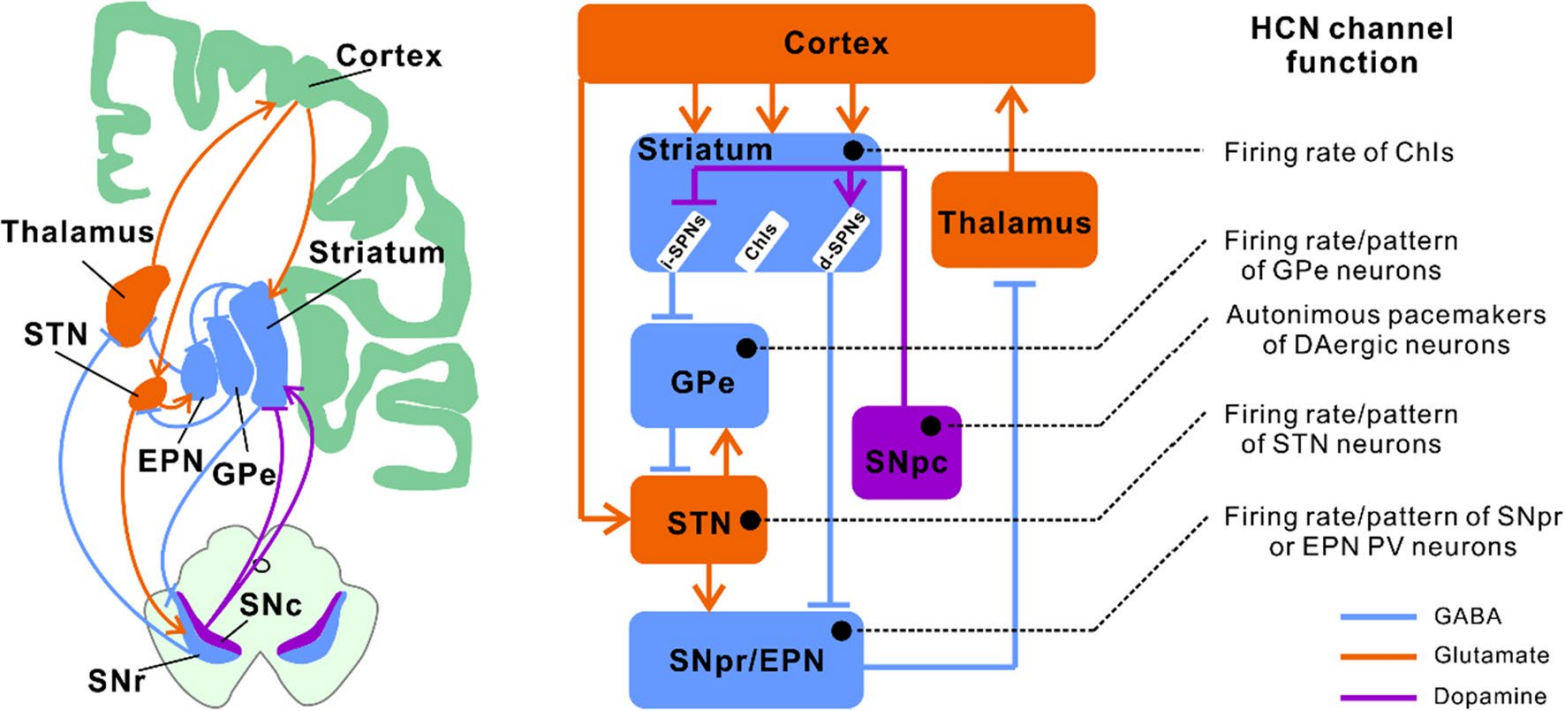
Function of neuronal HCN channels in basal ganglia nuclei. The basal ganglia, a subcortical structure, plays a crucial role in regulating motor function by processing information from and feeding it back to the cerebral cortex. Substantia nigra, a key nucleus within the basal ganglia, functions as a regulatory agent. HCN channels, which are important in regulating the normal electrical activity of neurons, are expressed in the basal ganglia nuclei. *HCN* hyperpolarization-activated cyclic nucleotide-gated channel, *ChIs* cholinergic interneurons, *d-SPNs* direct pathway-striatal projection neurons, *i-SPNs* indirect pathway-striatal projection neurons, *SNpc* substantia nigra pars compacta, *SNr* substantia nigra pars reticulata, *GPe* globus pallidus externus, *EPN* entopeduncular nucleus, *PV* parvalbumin, *STN* subthalamic nucleus

**Fig. 3:**
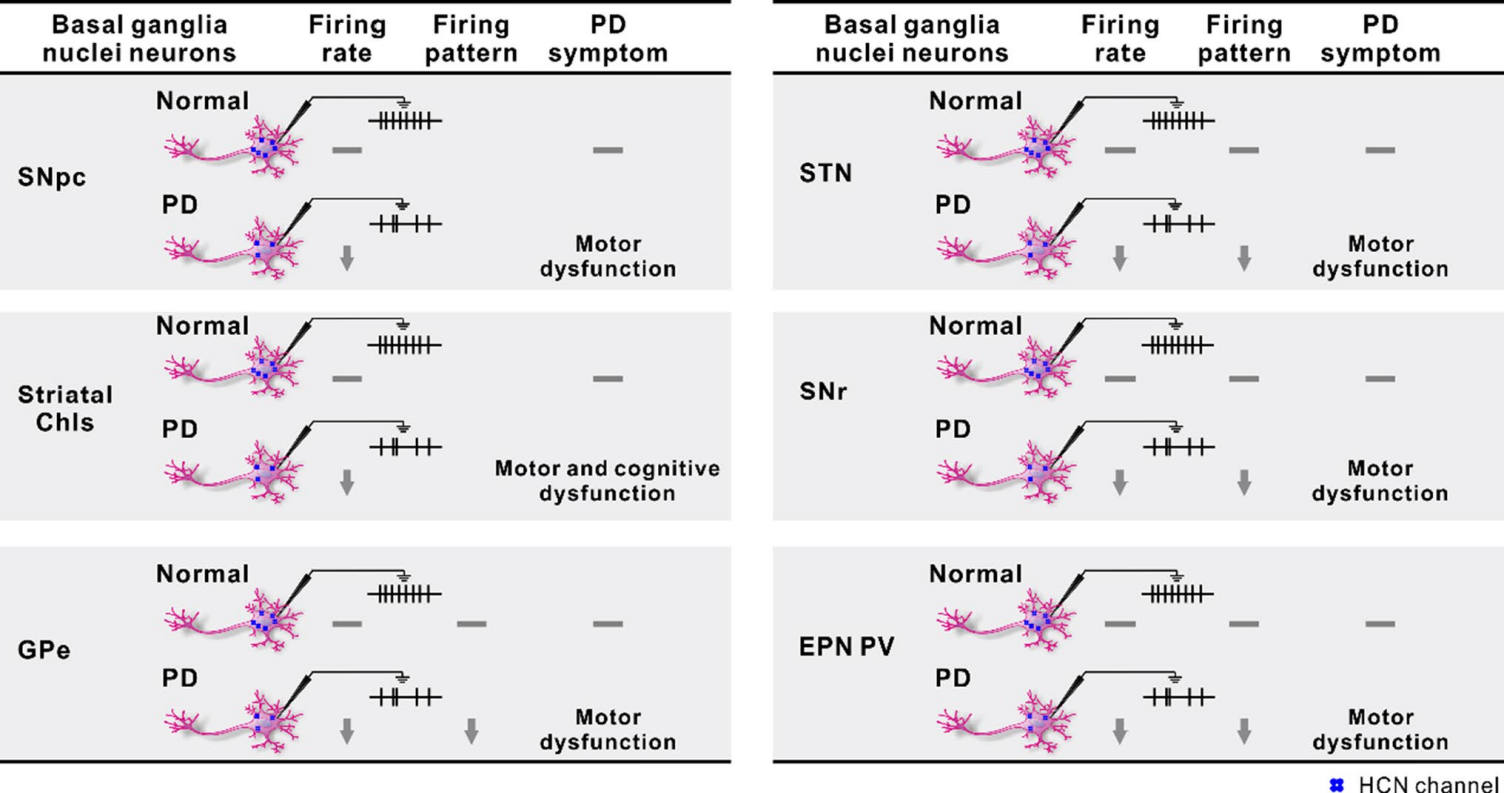
The relationship between the expression of HCN channels in the basal ganglia nuclei and the symptoms of PD. The expression of HCN channels in neurons of the basal ganglia nuclei was downregulated in the pathological state of PD. The downregulation of HCN channels in dopaminergic neurons results in decreased neuronal firing rates, which are associated with motor dysfunction. The downregulation of HCN channels in striatal ChIs results in decreased neuronal firing rates, which are associated with cognitive dysfunction. The downregulation of HCN channels in GPe/STN/SNr/EPN PV neurons results in decreased neuronal firing rates and irregularized firing pattern, which are associated with motor dysfunction. *PD* Parkinson’s disease, *HCN* hyperpolarization-activated cyclic nucleotide-gated channel, *ChIs* cholinergic interneurons, *SNpc* substantia nigra pars compacta, *SNr* substantia nigra pars reticulata, *GPe* globus pallidus externus, *EPN* entopeduncular nucleus, *PV* parvalbumin, *STN* subthalamic nucleus

The basal ganglia are integral to motor function regulation within the subcortex. PD, a serious motor disorder, results from an imbalance in the direct and indirect pathways of the basal ganglia due to dopaminergic neuron degeneration in the substantia nigra. Our recent studies have demonstrated the presence of HCN channels in various basal ganglia regions, including the striatum, globus pallidus (GP), and STN [18, 19, 21, 27]. It was observed that reduced expression of HCN channels correlates with PD pathology, impacting neuronal firing patterns and leading to movement disorders [19, 21]. Based on these findings and corroborating evidence from other research groups, we first reviewed the normal physiological function of HCN channels in basal ganglia nuclei neurons (Fig. 2) and then further reviewed the changes in the expression of HCN channels in PD pathology and their relationship with motor and non-motor symptoms (Fig. 3).

### HCN channels in the SNpc

PD is the second most prevalent neurodegenerative disorder, mainly characterized by the progressive deterioration of dopaminergic neurons in the SNpc [4, 57]. Multiple studies have attested to the association of dopaminergic phenotype with HCN expression. HCN channels are extensively expressed in basal ganglia nuclei neurons, including dopaminergic neurons of the SNpc [22], and the presence of the Ih current is a hallmark of midbrain dopaminergic neurons, including those of the SNpc [58]. The HCN channel of SNpc neurons is targeted by several neurotransmitters such as dopamine, serotonin, noradrenaline, neurotensin, and ghrelin. Dopamine activates the Gi-cAMP pathway by stimulating D2 receptors widely distributed in SNpc neurons, thereby suppressing adenylate cyclase activity and intracellular cAMP levels, ultimately reducing the amplitude of the Ih current on SNpc neurons. In addition, the perfusion of the brain slices from SNpc with serotonin decreases the Ih current, while noradrenaline significantly increases the Ih current in the SNpc neurons [30, 59]. Moreover, neurotensin caused a reversible decrease in the amplitude of Ih in the rat SNpc by reducing the maximum current; however, it did not change the voltage dependence of activation. The effect of nitric oxide (NT) depends on the activation of PKC pathways, as the NT-induced Ih inhibition was blocked by staurosporine, a specific PKC inhibitor, and mimicked using the PKC activator [43]. Furthermore, in PD, ghrelin increased the Ih current by activating the GHSR-adenylate cyclase (AC)-cAMP-PKA or PKC/RTK-MAPK pathway, counteracting Methyl-4-phenylpyridinium (MPP +)-induced Ih current inhibition, thus enhancing the electrical activity of dopaminergic neurons [60].

The HCN channel has been shown to modulate both RMP and the spontaneous firing activity of dopaminergic neurons, thereby affecting the firing activity of SNpc dopaminergic neurons by regulating cell membrane excitability [60, 61]. The RMP for dopaminergic neurons is usually within the range of -55 mV to -40 mV. When the neuron is hyperpolarized to -70 mV, the HCN channel opens and selectively allows Na^+^ and K^+^ inflow to maintain the cell membrane excitability [22, 30]. In addition, the HCN channel current Ih can regulate the reactivity of SNpc dopaminergic neurons to achieve excitatory synaptic transmission [62]. Early PD pathology may trigger mitochondrial failure and ATP depletion, resulting in the loss of Ih function [63]. This can then induce apoptosis of SNpc dopaminergic neurons through a calcium-dependent excitatory toxicity mechanism [64]. Dopaminergic neurons in the SNpc are “autonomous pacemakers” whose spontaneous electrical activity causes the continuous release of neuronal dopamine, maintaining its basal ganglia levels [65]. Studies have shown that the HCN channel on dopaminergic neurons essentially regulates dopamine release by maintaining the dopaminergic neuron’s spontaneous low-frequency firing after external signals [58, 66]. The discharge characteristics of dopaminergic neurons are linked to the pathogenesis of PD. The surviving dopaminergic neurons in the SNpc show alterations in discharge activity including a reduced spontaneous discharge frequency and number of discharging neurons, and an increase in sudden discharges [66]. At the same time, stereotactic administration of HCN channel blockers ZD7288 and ivabradine can lead to SNpc-specific neurodegeneration and to a semi-Parkinsonian motor phenotype in rats [23]. Thus, this evidence presented above strongly suggests that the adequate expression of HCN channels is crucial for maintaining proper neuronal function and electrical activity in the SNpc.

### HCN channels in the striatum

The striatum, the largest nucleus in the basal ganglia, regulates motor control, planning, procedure learning, and action selection. It consists mainly of GABAergic projection neurons, called striatal projection neurons (SPNs), which inhibit their targets when activated. The remaining neurons are mainly giant cholinergic (ChIs, 1%-3%) and GABAergic (2%-5%) interneurons [21, 67]. In PD, it has been shown that the striatum is particularly affected by the loss of SNpc dopaminergic neurons. Thus, the striatum is the primary therapeutic target for treating the condition [68]. In addition to dopaminergic innervation, it is widely believed that the large cholinergic interneurons in the striatum crucially regulate the movement by balancing dopamine signaling [69]. In individuals with PD, the hypercholinergic state can be determined by administering anticholinergic drugs, which can alleviate the motor symptoms of patients, while acetylcholinesterase inhibitors can worsen the symptoms, such as tremors and bradykinesia. Consequently, anticholinergic drugs were the standard treatment for PD prior to the emergence of levodopa [70]. The development of more precise surgical techniques has allowed the targeting of large cholinergic interneurons in the striatum and their associated receptors as a potential new treatment for PD [71].

The striatal ChIs express four HCN channel subtypes, with HCN2 and HCN3 as dominant subtypes that regulate the interneuron's discharge activity [72]. Progressive dopamine loss reduces the HCN mRNA transcription, decreases ChIs firing, and changes the structure of HCN channels. The reduction of HCN gene expression decreases the impact of dopamine on ChIs and changes motor function. The downregulation in gene expression of HCN channels could be due to a reduction in cAMP, PKA activity-dependent availability, due to the presence of the auxiliary HCN TRIP8B cytoplasmic protein, an alternative splicing isoform that contains helper HCN tetrapeptide repeats. The TRIP8b is modulates HCN surface expression and inhibition of CNBD activation through cAMP. Altering the extreme domain, which includes the C-linker and CBND domain, of the HCN channel can also change its functional association with TRIP8b, modifying channel expression and kinetics [17]. The HCN channel’s expression stabilization might improve the interaction between acetylcholine (ACh) and dopamine, thus enhancing motor function in PD patients[73]. Hence, decreased expression of HCN channels in ChIs may indicate midbrain dopaminergic neuronopathy during PD pathology [71]. Furthermore, it may indicate that reduced HCN channel activity in the striatum’s ChIs is essentially linked with PD development and related movement disorders. Prolonged treatment with l-3,4-dihydroxyphenylalanine (L-DOPA) can restore the HCN channel activity of ChIs. Although targeting HCN channels is an efficient therapeutic intervention, modulators that specifically act on the channels of each cell type to achieve effective and safe behavioral outcomes are still required [74].

Cognitive dysfunction in PD depends on the interaction of striatal ACh and dopamine systems [75, 76]. The lack of dopamine decreases HCN channel expression in the ChIs of the striatum, a reduction in Ih current, as well as decreased neuronal excitability, thus inhibiting ACh release [77]. Though both the production and release of Ach and dopamine are decreased, the concentration of ACh is still higher than that of dopamine, disrupting the balance between the two and leading to cognitive impairment [73]. Therefore, overexpressing HCN channels in ChIs may enhance the activity of these neurons, restoring the balance of the dopamine/ACh ratio and potentially ameliorating the cognitive dysfunction associated with PD.

### HCN channel in GPe

The GPe, also known as the lateral globus pallidus (LGP), plays a vital role in regulating the indirect pathway of the basal ganglia circuit, contributing to important motor regulation functions in both physiological and pathological circumstances [27, 78]. The GPe neuron subtypes are of two classes: the parvalbumin-expressing (PV^+^) neurons and the transcription factor Npas1-expressing (Npas1^+^) neurons. Approximately 55% of GPe are PV^+^ neurons, whereas only 27% are Npas1^+^ neurons. PV^+^ neurons primarily project to the STN, whereas Npas1^+^ neurons predominantly target the dorsal striatum [79]. Functionally, these two types of neurons act in unison to regulate the transitions between behavioral states in mice[80]. The HCN channel subtypes are widely distributed in GPe neurons, and, according to the results of single-cell q-PCR, LGP PV^+^ neurons express all four HCN channel subtypes. Among them, HCN2 channel subtype is the most highly expressed, followed by HCN1, HCN4, and HCN3 [27]. Immunoperoxidase labeling for HCN1 and HCN2 was observed in various GP regions, including somata, dendritic processes, myelinated and unmyelinated axons, and axon terminals [81]. Furthermore, the HCN channel also modulates synaptic transmission[82]. Perfusion of ZD7288 increased the frequency but not the amplitude of miniature inhibitory postsynaptic currents (mIPSCs) on GP neurons, implying that HCN channels regulate the presynaptic GABA release at these synapses [81].

PD is characterized by reduced dopamine that alters the firing patterns of GPe neurons. The electrophysiological and immunohistochemical experiments have indicated that GPe neurons receive dopaminergic innervation from axonal branches of SN-striatum fibers and that D1 and D2-like receptors are expressed in the GPe [83]. Dopamine mediates normal GPe neuronal pacing by upregulating the activity of HCN channels, which in turn depolarizes the neuron. Reduced dopamine levels decrease the depolarizing effect of the HCN channel. Furthermore, the expression of HCN channels is inhibited, leading to a decrease in the autonomous pacemaker function of the GPe neuron due to a lack of dopamine in PD. [20, 84]. In addition, in line with the HCN channel downregulation that controls pacemaker activity of pallidal neurons in PD-afflicted animals, administration of intra-pallidal ZD7288 injection diminished the proportion of slow-firing neurons in the pallidum, subsequently improving the locomotor activity in MPTP parkinsonian mice [84]. Moreover, The HCN channel is essential for sustaining the firing pattern of LGP neurons, and continuous depletion of dopamine decreases the current of the HCN channel in GPe neurons[85]. Concurrently, the mRNA and protein levels of the four pore-forming HCN subunits and the HCN transport protein TRIP8b were reduced [30]. Downregulation of the HCN2 subunit, dominant in LGP, is the most significant alteration. The selective knockdown of the gene encoding HCN2 silences LGP neurons. It was observed that the viral delivery of the HCN2 expression constructs reversed the firing activity loss after dopamine depletion and regularized the firing patterns of neurons [20]. Demonstrating that HCN2 downregulation inhibits neuronal firing activity and regularizes the firing patterns. Alterations to firing rates and patterns have been consistently linked to motor symptoms of PD in animal models and human patients, such as bradykinesia, static tremor, gait instability, and rigidity; moreover, these are also closely related to the decreases in LGP neuron activity and increased synchronous oscillatory discharge activity due to dopamine loss [20, 80].

### HCN channel in the STN

The STN is the only excitatory glutamatergic nucleus in the basal ganglia circuit, serving as an important node in the classical indirect pathway. It receives direct projection from the cortex and forms a hyper-direct pathway, acting as a pacemaker that modulates all basal ganglia circuit activities [19, 86]. In the classical indirect pathway, the STN receives GABAergic inputs from the GPe and sends glutamatergic nerve fibers to the GPi, increasing the inhibitory output of the basal ganglia from the thalamus and terminating motor behavior resulting from the activation of the direct pathway. This pathway balances the indirect and direct pathways, maintaining the normal motor function of the basal ganglia circuit [87, 88]. Recently, it has been revealed that the hyper-direct glutamatergic pathway from the cerebral cortex to STN and the classical indirect pathway through STN functionally inhibit movement [31]. In PD, the basal ganglia lose its dynamic regulation of dopaminergic afferent neurons in the SN, causing a relative imbalance between the hyper-direct and indirect pathways. Increased dysfunction of basal ganglia circuitry and activity becomes a significant characteristic of PD, ultimately contributing to motor impairment in patients [89, 90].

The presence of the four HCN channel subtypes in STN neurons was confirmed via single-cell q-PCR, immunohistochemistry, and western blot analyses. The HCN channel activity closely relates to the firing activity of STN neurons and motor behavior [19, 91]. With the help of multi-barrel extracellular recordings, *in-vivo* bidirectional modulation of STN neuron firing rates was assessed by the selective HCN channel blocker ZD7288 and agonist 8-Br-cAMP through micro-pressure ejection. The unilateral microinjection of ZD7288 or 8-Br-cAMP was administered to acquire postural behavior in conscious rats [91]. Additionally, the firing patterns of STN neurons were regularized through pharmacological activation of HCN channels, whereas its inhibition disrupted their firing patterns [19]. Furthermore, the haloperidol ZD7288 or 8-Br-cAMP unilaterally administered in the STN indicated significantly deviant posture on the contralateral or ipsilateral side [91].

The STN firing pattern configurations may determine the pathogenesis of PD symptoms and provide insight into the normal motor control mechanisms [19, 92–94]. The STN exhibits excessive firing bursts in animals deprived of dopamine, suggesting that the firing pattern is a crucial pathophysiological component causally related to the locomotor deficits observed in PD. STN burst discharge alterations via techniques such as DBS of different polarities reduce or increase the associated locomotor deficits [92, 93]. In addition, during *in-vivo* experiments on freely moving rats, the local injection of an HCN blocker in the STNs resulted in a higher power of high-voltage spindles (HVSs), which are characteristic oscillations with theta frequencies and are linked with immobility behavior. Moreover, HVSs have displayed an increased power during PD. Interestingly, the effect of the HCN blocker was reversed by the local injection of lamotrigine, an HCN agonist [94]. Furthermore, in a PD rat model it has been shown that the HCN channel expression in the STN neurons of a PD rat model is closely linked to the neuronal firing mode. Thus, it may be logical to suppose that during PD pathogenesis, HCN channel expression in STN neurons is reduced, therefore disrupting neuronal firing pattern and leading to movement disorders. Therefore, increasing HCN2 channel expression in STN neurons can regulate the neuronal firing patterns and improve the dyskinesia of animals [19].

### HCN channels in substantia nigra pars reticulata (SNr)

The SNr mainly comprises neurons that utilize gamma-aminobutyric acid (GABA) as their neurotransmitter. It is the primary output nucleus of the basal ganglia and integrates information from other basal ganglia nuclei (striatum, GPe, and STN) and transmits it to external structures, regulating processes such as motor, cognitive, and emotional-motivational. Electrophysiology tissue and single-cell RNA sequencing have revealed the distribution of HCN channels in SNr [15, 95, 96]. The SNr GABAergic neurons within the SN microcircuit exhibit an Ih current with plateau potential and significant depression amplitude. Furthermore, there are variations in the Ih current amplitude among SNr GABAergic neurons. By enhancing the HCN conductance of SNr GABAergic neurons, a higher Ih current amplitude can modulate the GABAergic input received by SNpc dopaminergic neurons, suggesting that HCN channels may indirectly influence the activity of SNr GABAergic neurons [95]. Additionally, the stimulation of GPe GABAergic neurons, which project to SNr, activates HCN channels in SNr GABAergic neurons. Through this mechanism, the currents activated by hyperpolarization show SNr responses that are excitatory and biphasic inhibitory-to-excitatory. This suggests that the HCN channel also regulates the excitability of SNr GABAergic neurons [96].

The function of HCN channels in SNr neurons is closely associated with the neuronal firing rate and pattern. About 50% of SNr neurons in dopamine-depleted mice exhibited delta oscillations, which were related to alterations in discharge rate, irregularity, bursts, and synchronicity [97]. Delta oscillation may be related to the interaction between the HCN channels in hyperpolarized neurons [98]. Moreover, the absence of dopamine reveals pathological central pattern generators (CPGs) that exhibit an unusual number of neurons involved in rhythmic bursting in SNr [99]. ZD7288, an HCN channel antagonist, inhibited the inward current conducted through HCN channels, causing increased after hyperpolarizations (AHPs) that follow the bursts. Consequently, it decreased neuronal firing rate and regularity. During the interburst intervals (IBIs), outward currents are presumed to dominate, suggesting that a mixture of HCN channels forms IBIs [100]. Therefore, the HCN channels in SNr neurons may contribute to the bursting firing pattern.

### HCN channel in GPi

The GPi (entopeduncular nucleus (EPN) in rodents) is the primary output core of the basal ganglia. It is believed to receive and integrate information from the direct and indirect basal ganglia pathways and send it to various functional targets to integrate motion information and control the precise execution of motion programs [101]. EPN comprises at least four types of GABAergic neurons; of these, around 29% are positive for PV and are primarily found in the caudal/posterior two-thirds of the central nuclear EPN, and approximately 6.8%, 38.9%, and 20.1% are somatostatin (SST) and nitric oxide synthase (NOS)-expressing neurons, SST-only neurons, and NOS-only neurons, respectively. In EPN, these neurons are located in the rostral/anterior half and the shell region [102, 103]. It has been indicated that the EPN has a critical function in different physiological and pathological processes, such as controlling movements in PD. For example, injecting botulinum toxin type A into the EPN alleviates the extent of gait rigidity in animal PD models [104]. Furthermore, research indicates that GPi is an essential deep brain stimulation (DBS) target for PD [105, 106]. GPi/EPN stimulation through DBS has the potential to alleviate generalized dystonia in PD patients and animal models [107].

In the mammalian brain, HCN channels are widely expressed throughout most basal ganglia nuclei, including the EPN [30, 72]. According to single-cell q-PCR experiments, all four HCN channel subtypes are expressed in EPN PV^+^ neurons with the highest expression levels of the HCN2 subtype, followed by HCN1, HCN4, and HCN3. The HCN channels maintain the RMP of EPN PV^+^ neurons and can influence their excitability and firing mode. Pharmacological activation of the HCN channel on EPN PV^+^ neurons increase the channel's half-activating potential and activation time constant. Moreover, selective HCN2 channel downregulation decreases the excitability and RMP of PV^+^ neurons in EPN [18]. Animal PD models show changes in the expression and function of HCN channel subtypes in the EPN, suggesting a correlation between the GPi neuron’s HCN channel activity and the development and occurrence of PD [18]. Furthermore, in the mouse PD model, the decrease in expression of EPN PV^+^ neuron’s HCN channel subtypes resulted in a slower neuronal firing rate and an irregular firing pattern, which caused motor dysfunction characteristics of PD. Moreover, CRISPR/Cas9-induced HCN2 down regulation in EPN PV^+^ caused irregularities in neuronal firing patterns and aggravated motor dysfunction in mouse models of PD; however, its upregulation produced regularity in neuronal firing patterns and alleviated motor dysfunction [18].

## Microglial HCN channel involved in PD neuroinflammation

Neuroinflammation in PD, characterized by microglial activation, contributes to dopaminergic neuron degeneration and disease progression [108]. Notably, the substantia nigra exhibits higher microglial density than other brain regions, increasing the vulnerability of dopaminergic neurons to external immune stimuli. Furthermore, systemic or intracerebral lipopolysaccharide injections significantly increase microglial proliferation and decrease the number of tyrosine hydroxylase-positive neurons in the substantia nigra [108].

The central nervous system indicates signs of persistent inflammation during early PD stages, and the HCN channel is among the most widely characterized channel proteins involved in neuroinflammation [109]. The literature suggests that HCN channels may contribute to PD pathogenesis by affecting the neuroinflammatory response. Microglia are the main type of glia involved in the brain’s inflammatory response [110]. When a neurotoxin, such as MPP^+^, persists in the brain and exceeds the microglial compensatory capacity, microglia are activated to play an anti-inflammatory role, leading to dopaminergic neuronal deformation and necrosis through phagocytosis [110, 111]. Activated microglia and increased inflammatory cytokines release (IL-1β and TNF-α) have been observed in the SNpc and striatum of PD patients and PD animal models [112]. In a recent study, Vay et al*.* confirmed for the first time that microglia express all HCN channel subtypes and that microglial HCN channel expression changes after pro-inflammatory and anti-inflammatory stimuli, where HCN2 channel expression is upregulated during pro-inflammatory stimulation and downregulated during anti-inflammatory stimulation, while that of HCN3 is downregulated under both stimuli. In addition, blocking or silencing HCN2 channels can inhibit microglial activation [34], and since ZD7288 blocks the HCN channel, it inhibits microglial proliferation [113]. Since neuroinflammation is a characteristic feature of various neurological disorders, microglial HCN channel activity could serve as a novel therapeutic target in the treatment of PD [34].

## Conclusion

Recent evidence indicates that HCN channels in the basal ganglia nucleus neurons may serve as promising targets for treating PD. In the absence of disease, the expression of HCN channels in each basal ganglia nucleus plays a crucial role in maintaining normal basal ganglia physiological functions. HCN channel expression in the basal ganglia nuclei decreases during PD onset and development. This reduction impacts neuronal firing rates and patterns, leading to PD's motor and non-motor symptoms. Pharmacological activation or overexpression of specific HCN channel subtypes may alleviate the symptoms of PD. Targeting HCN channels or their specific subtypes with drugs or gene therapies presents a significant potential for clinical PD treatment. Modulating HCN channels in microglia, which are involved in PD's inflammatory response, could potentially reduce inflammation and protect dopaminergic neurons. Further research into the relationship between changes in HCN and its regulatory proteins in the basal ganglia and PD symptoms is crucial for future investigation.

## Data Availability

Not applicable.

## Abbreviations

PD: Parkinson’s disease
HCN: Hyperpolarization-activated cyclic nucleotide-gated channel
Ih: Hyperpolarization-activated current
LC: Locus ceruleus
SNpc: Substantia nigra pars compacta
SNr: Substantia nigra pars reticulate
GWAS: Genome-wide association studies
GP: Globus pallidus
GPe: Globus pallidus externus
GPi: Globus pallidus internus
LGP: Lateral globus pallidus
EPN: Entopeduncular nucleus
STN: Subthalamic nucleus
MPTP: 1-Methyl-4-phenyl-1,2,3,6-tetrahydropyridine
CAD: Chronic coronary artery disease
CHF: Chronic heart failure
AD: Alzheimer's disease
ALS: Amyotrophic lateral sclerosis
SMA: Spinal muscular atrophy
RMP: Resting membrane potential
CNBD: Cyclic nucleotide-binding domain
GYG: Glycine-tyrosine-glycine amino-acid
LHb: Lateral habenular nucleus
MSO: Medial superior olive
DRG: Dorsal root ganglion
NT: Nitric oxide
MPP: Methyl-4-phenylpyridinium
CPGs: Central pattern generators
AHPs: After hyperpolarizations
IBIs: Interburst intervals
SPNs: Striatal projection neurons
d-SPNs: Direct pathway-striatal projection neurons
i-SPNs: Indirect pathway-striatal projection neurons
ChIs: Cholinergic interneurons
TRIP8b: Tetratricopeptide repeat-containing Rab8b-interacting protein
L-DOPA: L-3,4-dihydroxyphenylalanine
PV: Parvalbumin
mIPSCs: Miniature inhibitory postsynaptic currents
EPN: Entopeduncular nucleus
SST: Somatostatin
NOS: Nitric oxide synthase
DBS: Deep brain stimulation
HVSs: High-voltage spindles
ACh: Acetylcholine
HEK293: Human embryonic kidney 293
MSO: Medial superior olive
BCs: Basket cells
